# *Cryptochrome-1* Gene Expression is a Reliable Prognostic Indicator in Egyptian Patients with Chronic Lymphocytic Leukemia: A Prospective Cohort Study

**DOI:** 10.4274/tjh.2017.0169

**Published:** 2018-08-05

**Authors:** Deena Mohamed Habashy, Deena Samir Eissa, Mona Mahmoud Aboelez

**Affiliations:** 1 Ain Shams University Faculty of Medicine, Department of Clinical Pathology, Unit of Hematology, Cairo, Egypt

**Keywords:** Chronic lymphocytic leukemia, Cryptochrome-1, Circadian genes, Time to first treatment, Prognosis, Real-time polymerase chain reaction, CD38, Zap-70

## Abstract

**Objective:**

Traditional prognostic factors have proved insufficient to account for heterogeneity in the clinical behavior of chronic lymphocytic leukemia (CLL). *Cryptochrome-1* (*CRY-1*) is a circadian clock gene essential in maintaining the circadian rhythm and regulating cell proliferation. We evaluated *CRY-1* gene expression in CLL and addressed its putative role as a prognostic indicator for the clinical course of CLL.

**Materials and Methods:**

A total of 100 CLL patients at diagnosis were studied for *CRY-1* gene expression by real-time reverse-transcription polymerase chain reaction and were followed for assessment of time to first treatment (TFT).

**Results:**

*CRY-1* was expressed in 94% of the CLL patients at diagnosis. The median *CRY-1* relative gene expression level (0.006) stratified patients into high and low expression groups. Forty of 100 (40%) CLL patients showed high *CRY-1*, 54/100 (54%) showed low *CRY-1*, and 6/100 (6%) had undetectable *CRY-1* gene expression. High *CRY-1* gene expression was concordant with CD38^+^, Zap-70^+^, and double CD38^+^Zap-70^+^ expression; unfavorable/intermediate cytogenetics; unmutated *immunoglobulin heavy-chain variable-region* gene; and diffuse marrow infiltration. The high *CRY-1* gene expression patient group exhibited shorter TFT than the patients with low *CRY-1* gene expression. A Cox proportional hazard regression model identified *CRY-1* gene expression to be independently predictive for TFT.

**Conclusion:**

*CRY-1* is differentially expressed among CLL patients, stratifying them into low-risk and high-risk groups. *CRY-1* gene expression could constitute a reliable prognostic indicator for CLL progression, complementing the role of standard well-established prognostic factors. *CRY-1* gene expression could be employed as a prognostic indicator for disease progression during the initial prognostic work-up and follow-up for CLL patients.

## Abstract (tr)

**Amaç:**

Geleneksel prognostik faktörler, kronik lenfositik löseminin (KLL) klinik davranışındaki heterojenitenin nedenini açıklamak için yetersiz bulunmaktadır. *Kriptokrom-1* (*CRY-1*) sirkadiyen ritmin sağlanması ve hücre proliferasyonu regülasyonunda gerekli olan bir sirkadiyen saat genidir. Biz, KLL’de *CRY-1* gen ekspresyonunu değerlendirdik ve KLL’nin klinik seyri için prognostik gösterge olarak varsayılan rolünü irdeledik.

**Gereç ve Yöntemler:**

Toplam 100 KLL hastası tanı sırasında kantitatif gerçek zamanlı ters transkripsiyon polimeraz zincir reaksiyonu ile *CRY-1* gen ekspresyonu için incelendi ve ilk tedaviye kadar geçen zamanın (TFT) değerlendirilmesi için takip edildi.

**Bulgular:**

KLL hastalarının %94’ünde tanı anında *CRY-1* ekspresyonu mevcuttu. Medyan CRF-1 rölatif gen ekspresyon düzeyi (0,006) hastaları yüksek ve düşük ekspresyon gruplarına ayırmaktadır. Yüz hastanın 40’ı (%40) yüksek *CRY-1*, 54/100’ü (%54) düşük *CRY-1*, ve 6/100’ü (%6) tespit edilemez *CRY-1* gen ekspresyonu gösterdi. Yüksek *CRY-1* gen ekspresyonu CD38^+^, Zap-70^+^ ve çift CD38^+^Zap-70^+^ ekspresyonu; olumsuz/orta sitogenetik; mutasyona uğramamış *immünoglobulin ağır zincir değişken bölge* geni ve diffüz kemik iliği infiltrasyonu ile uyumlu idi. Yüksek *CRY-1* gen ekspresyonu olan hasta grubu, düşük *CRY-1* gen ekspresyonu olan hastalardan daha kısa TFT gösterdi. Cox oransal hazard regresyon modeli *CRY-1* gen ekspresyonunu TFT için bağımsız prediktif olarak tanımlamıştır.

**Sonuç:**

*CRY-1*, KLL hastalarında farklı olarak eksprese edilerek onları düşük-risk ve yüksek-risk gruplarına ayırmaktadır. *CRY-1* gen ekspresyonu, KLL progresyonu için iyi-belirlenmiş standart prognostik planlamada ve KLL hastalarının takibinde hastalık progresyonu için prognostik bir gösterge olarak kullanılabilir.

## Introduction

Chronic lymphocytic leukemia (CLL) is a lymphoproliferative neoplasm defined by proliferation and accumulation of morphologically mature, immunologically dysfunctional monoclonal B cells in the peripheral blood (PB), bone marrow (BM), and lymphatic tissues [1].

The clinical course of CLL is heterogeneous and difficult to predict; some patients may exhibit rapid disease progression while others may live for decades without requiring treatment [2]. Early treatment of the latter group would risk the development of therapy-related complications that might affect the quality of life and/or survival. Assigning markers that reliably stratify patients into good-risk or poor-risk disease groups could help in evaluating the potential benefit of early treatment and assist risk-adapted treatment strategies [3]. Traditional prognostic factors have proved insufficient to account for heterogeneity in the clinical behavior of CLL, indicating a need for further prognostic indicators that can better correlate with patients’ clinical outcome and survival [4,5].

The *immunoglobulin heavy-chain variable-region *(*IgHV*) gene mutational status correlates with clinical behavior and represents a robust prognostic indicator for CLL. However, *IgHV* gene sequencing is complicated and time-consuming for routine laboratories; hence, exploring an alternative for *IgHV* gene mutations is a priority [6].

The circadian machinery comprises various genes that show functional interplays with cell cycle regulators. Aberrant expression of circadian clock genes can therefore lead to aberrant expression of downstream target genes responsible for cell proliferation and apoptosis, resulting in the emergence of different types of cancers, including CLL [6,7,8,9].

The *Cryptochrome-1*(*CRY-1*) gene (12q23-q24.1) is a member of the circadian clock essential to the maintenance of circadian rhythm. In addition to its circadian function, it also acts as a transcriptional regulator for several genes with roles in cell metabolism and proliferation [10].

Accordingly, we aimed to evaluate *CRY-1* gene expression in CLL patients and address the gene’s putative role as a prognostic indicator for the clinical course of CLL.

## Materials and Methods

This prospective cohort study included 100 newly diagnosed and untreated CLL patients who attended the Hematology/Oncology Clinic of the Ain Shams University Hospitals. Patients were selected on the basis of standard clinical, hematologic, and immunophenotypic characteristics for the diagnosis of CLL [11]. These CLL patients comprised 64 males and 36 females; the male to female ratio was 1.8:1 and median age was 61 years (interquartile range (IQR): 55-68 years). All patients were followed after diagnosis for assessment of the time to first treatment (TFT) (median: 20 months; range: 10-24 months).

TFT is the time from diagnosis to the start of therapy or the last follow-up. The criteria for starting treatment are: (i) the existence of constitutional symptoms; (ii) progressive marrow failure indicated by anemia and/or thrombocythemia; (iii) autoimmune anemia and/or thrombocytopenia not responding to steroids; (iv) massive (more than 6 cm below the costal margin) or progressive splenomegaly; (v) massive nodes or nodal clusters (more than 10 cm in the longest diameter) or progressive lymphadenopathy; (vi) progressive lymphocytosis by more than 50% over 2 months or a doubling time of less than 6 months [12]. Informed consent was provided prior to patient enrollment. The study protocol was endorsed by the Ethical Committee for Human Research of Ain Shams University and complied with the Helsinki Declaration of 1975, as revised in 2002. The clinicopathologic characteristics of CLL patients at diagnosis are presented in Table 1.

### Sampling

PB and BM samples were collected in K-EDTA (1.5 mg/mL) for morphologic, immunophenotypic, and molecular analyses and in lithium heparin for cytogenetic analysis. PB samples were used for flow cytometric immunophenotyping and real-time reverse-transcription polymerase chain reaction (qRT-PCR) for quantification of *CRY-1* gene expression, while PB or BM samples, when available, were used for the cytogenetic analysis.

### Flow Cytometric Immunophenotyping

Immunophenotyping using the standard panel for chronic lymphoproliferative disease (CD5, CD19, CD23, FMC7, CD20, CD38, CD79b, CD10, CD25, CD103, CD123, kappa, lambda, surface immunoglobulin G) (Beckman Coulter, Miami, FL, USA), along with ZAP-70 (BioLegend, San Diego, CA, USA), was performed with the EPICS XL Flow Cytometer (Coulter Electronics, Hialeah, FL, USA). The positivity threshold was defined as the expression of the marker by ≥30% of the B lymphocytes [13]; however, for CD38 and Zap-70 a cut-off value of ≥20% and ≥10% of the B lymphocytes was considered positive, respectively [14].

### Fluorescence In Situ Hybridization

Probes for 13q-, 17p-, 11q-, and +12 (Vysis, Downers Grove, IL, USA) were used. Patients were stratified into cytogenetic-based risk groups as follows: favorable, 13q- or normal karyotype; intermediate, +12; and unfavorable, 17p-, 11q-, or complex karyotype (≥3 chromosomal aberrations) [13].

### qRT-PCR for Quantification of *CRY-1* Gene Expression

qRT-PCR amplification was done using gene expression sets for the *CRY-1* gene (*Homo sapiens*; Hs00172734-m1 TaqMan^®^ Gene Expression Assays), TaqMan b*-actin* control reagents for the b*-actin* reference gene, and TaqMan Universal PCR Master Mix (all from Applied Biosystems, Foster City, CA, USA). Total RNA was extracted from PB samples using a QIAamp^®^ RNA blood kit (QIAGEN, Valencia, CA, USA), while cDNA was synthesized using a QuantiTectReverse Transcription Kit (Applied Biosystems) according to the manufacturer’s protocol. The cDNA was stored at -20 °C until it was used.

cDNA samples were employed in synthesizing PCR products using sequence-specific primers and TaqMan oligonucleotide fluorescence-labeled probes: FAM for the *CRY-1* gene (Hs_*CRY-1*_1_FAM Quantifast Probe Assay, QIAGEN) and MAX for the b*-actin* gene (Hs_b-*actin*_1_MAX Quantifast Probe Assay, QIAGEN). Both probes were labeled with Iowa Black Fluorescent Quencher. Each PCR included all the necessary reagents and 50 ng of cDNA in a final volume of 25 µL. A negative control (cDNA replaced by nuclease-free water) was included in each assay. The reaction protocol comprised 40 cycles of heating at 95 °C for 5 min (hot-start PCR), then heating at 95 °C for 30 s (denaturation), and then heating at 60 °C for 30 s (annealing/extension). PCR and data analysis were carried out with Stratagene Mx3000P (Stratagene Inc., La Jolla, CA, USA). Undetectable *CRY-1* was noted for cases in which cycle threshold (CT) values exceeded the 40^th^ cycle.

*CRY-1* expression levels in unknown samples were calculated by relative quantification using the ΔΔCT method, which relies on comparison of CT values of *CRY-1* (target gene) to b*-actin* (reference gene) in unknown and normal calibrator samples. The results were expressed as the fold change in gene expression normalized to the reference gene and relative to the calibrator [15].

### Statistical Analysis

Analysis of data was done using SPSS 17 for Windows 7 (SPSS Inc., Chicago, IL, USA). Categorical variables are presented as frequency and percentage, compared using the chi-square (χ^2^) test. Continuous variables are presented as mean ± standard deviation or median and IQR for parametric and nonparametric variables, respectively. Student and Mann-Whitney U tests were employed for comparing continuous parametric and nonparametric variables between two groups, respectively. Kaplan-Meier curves for TFT were plotted and the log-rank test was used to compare TFT distributions between high and low *CRY-1 *gene expression groups. Multivariate Cox proportional hazard regression analysis (hazard ratio, HR) was employed to identify the independent association of *CRY-1* gene expression with TFT. Values of p<0.05 and p<0.01 were taken to be significant and highly significant, respectively.

## Results

### *CRY-1* Gene Expression in CLL Patients at Diagnosis

**Figure 1:**
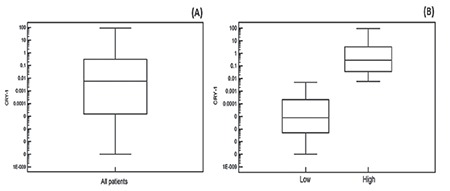
*Cryptochrome-1* gene expression in chronic lymphocytic leukemia patients at diagnosis: (A) all chronic lymphocytic leukemia patients, (B) low and high *Cryptochrome-1* gene expression groups. *CRY-1: Cryptochrome-1*.

The *CRY-1*gene was expressed in 94 of 100 (94%) CLL patients at diagnosis (median: 0.006; IQR: 0.000008-0.32) (Table 1). The median *CRY-1 *relative gene expression level (0.006) was employed as the cut-off value for stratifying high and low *CRY-1* gene expression groups. Accordingly, 40 of 100 (40%) CLL patients showed high *CRY-1 *gene expression (≥0.006) (median: 0.295; IQR: 0.034-3.618) and 54 of 100 (54%) CLL patients showed low *CRY-1* gene expression (<0.006) (median: 0.000008; IQR: 0.0000005-0.0002). Six of 100 (6%) CLL patients had undetectable *CRY-1* expression (Table 1, Figure 1).

### High and Low *CRY-1* Gene Expression and Clinicopathologic Characteristics of CLL Patients at Diagnosis

High *CRY-1* gene expression was significantly related to CD38^+^, Zap-70^+^, and double CD38^+^Zap-70^+^ expression; unfavorable/intermediate cytogenetics; unmutated *IgHV* gene; and diffuse BM infiltration in trephine biopsy (p<0.05). On the contrary, low *CRY-1* gene expression was significantly related to CD38^-^, Zap-70^-^, and double CD38^-^Zap-70^-^ expression and favorable cytogenetics (p<0.05). No further significance was found between high and low *CRY-1* groups regarding other studied clinicopathologic parameters (p>0.05) (Table 2).

### *CRY-1* Gene Expression and TFT of CLL Patients

**Figure 2:**
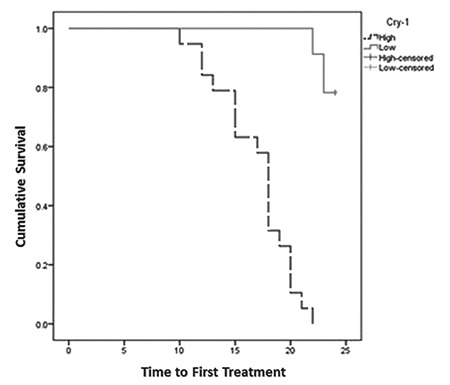
Kaplan-Meier curve showing the time to first treatment for chronic lymphocytic leukemia patients based on *Cryptochrome-1* gene expression levels. *CRY-1: Cryptochrome-1*.

Using the Kaplan-Meier method, the TFT for the high *CRY-1* gene expression group (median: 16.89 months; 95% confidence interval (CI): 15.38-18.41) was found to be significantly shorter than the TFT for the low *CRY-1* gene expression group (median: 23.69 months; 95% CI: 23.44-23.95) (p<0.001) (Table 2, Figure 2).

Multivariate Cox hazard regression analysis denoted *CRY-1* gene expression to be independently predictive for TFT (HR: 3.99; 95% CI: 2.12-6.19; p=0.001). The same was also shown for *IgHV* mutational status (p<0.001), Binet stage, Zap-70, and cytogenetic-based risk groups (p<0.05) (Table 3).

## Discussion

Diverse circadian genes are known to be correlated with poor prognosis when aberrantly overexpressed in several cancers, including *CRY-1 *[16]. In this work, the *CRY-1* gene was expressed in 94% of the studied CLL patients at diagnosis. The median relative* CRY-1* gene expression level (0.006) was employed to stratify patients into high and low expression groups. High *CRY-1* was related to CD38^+^, Zap-70^+^, and double CD38^+^Zap-70^+^ expression while low *CRY-1* was associated with CD38^-^, Zap-70^-^, and double CD38^-^Zap-70^-^ expression and discordant CD38/Zap-70 was comparable between the high and low *CR*Y-*1* gene expression groups. Similarly, a significant concordance was previously shown between elevated *CRY-1* transcripts and high-risk patients, as defined by CD38^+^ and/or unmutated *IgHV* genes, compared with their low-risk counterparts, as defined by CD38^-^ and/or mutated *IgHV* genes [6,10,17]. Moreover, elevated *CRY-1* transcripts in high-risk patients were found to be associated with Zap-70^+^ and double CD38^+^Zap-70^+^ expression [6,17].

Unlike our findings, cases of discordant CD38/Zap-70 expression (intermediate-risk) showed *CRY-1* gene expression levels comparable to those of the high-risk group in a previous study [17]. This discrepancy might be attributed to two major pitfalls that could hamper the prognostic use of CD38: first, the debate about the threshold that indicates CD38^+^ expression for defining patient prognosis, and second, the probability that CD38 expression may be unstable and differ over time, provoking the concern that CD38 may be an unreliable marker in CLL [18]. Another possible explanation is the variation in the positivity threshold for Zap-70 in the literature [6,14,17]. In accordance with Deaglio et al. [14], we used a cut-off value of ≥10% to indicate Zap-70^+^ expression. The threshold values for CD38 and Zap-70 used by Deaglio et al. [14] were functional thresholds, below which CD38-mediated Zap-70 tyrosine phosphorylation was undetectable.

We found an association between high *CRY-1* gene expression and both unfavorable/intermediate cytogenetic abnormalities and diffuse BM infiltration. In accordance, high *CRY-1* gene expression was detected in association with 17p- and +12, while low *CRY-1* gene expression was found in a case with 13q- as the sole abnormality [6]. Interestingly, since the *CRY-1* gene is located at chromosome 12q23-q24.1, an increased copy number was detected in patients with +12 [10]. On the other hand, we could not find further association between *CRY-1* gene expression levels and other studied clinicopathologic parameters. Yu et al. [19] also stated that neither age nor sex was associated with *CRY-1* gene expression level.

In our study, *CRY-1* was differentially expressed among CLL patients with mutated and unmutated *IgHV* genes, being overexpressed in the unmutated group. Other authors advocated the association between high *CRY-1* gene expression and the unmutated *IgHV* gene [6,10,17]. A cut-off value of 0.090 for *CRY-1* gene expression in CD19^+^ B cells of CLL patients was previously determined [6] and considered the best cut-off for segregating patients with mutated and unmutated *IgHV* genes (sensitivity: 95%; specificity: 92%; area under the curve: 0.963). The data of that study showed 92.8% concordance between *CRY-1* expression and *IgHV* mutational status.

We observed a shorter median TFT in the group with high *CRY-1 *expression (16.89 months) compared with that with low *CRY-1 *gene expression (23.69 months). Lewintre et al. [6] reported that high *CRY-1* gene expression was significantly related to shorter median progression-free survival of 63.2 months (95% CI: 48.2-78.2), compared to a median of 139 months (95% CI: 133.1-146.4) for the low *CRY-1* gene expression group (p<0.0001). Using multivariate Cox hazard regression, we found that *CRY-1* gene expression was independently predictive for TFT (p=0.001). Eisele et al. [17] employed univariate Cox hazard regression and reported that *CRY-1* could predict the clinical outcome of CLL patients as measured by TFT.

Of note, Hanoun et al. [10] reported that the methylation status of the *CRY-1* promoter revealed a considerable prognostic influence in CLL, whereby patients with hypermethylated *CRY-1* promoters showed significantly longer treatment-free survivals compared with their hypomethylated counterparts. Unexpectedly, they reported comparable levels of *CRY-1* mRNA in high-risk CLL and normal donor B cells. Thus, they postulated that expression differences of the *CRY-1* gene in CLL could be attributed to an underexpression of *CRY-1* in low-risk cases of CLL rather than an overexpression in the high-risk group.

## Conclusion

The circadian clock gene *CRY-1* is differentially expressed among CLL patients, stratifying them into low-risk and high-risk groups. *CRY-1* gene expression could constitute a reliable prognostic indicator for CLL progression, complementing the role of standard well-established prognostic factors. Accordingly, *CRY-1* gene expression could be employed as a prognostic indicator for disease progression during the initial prognostic work-up and follow-up for CLL patients.

Evaluation of *CRY-1* expression with respect to the overall survival of CLL patients is warranted. Study of *CRY-1* gene methylation status and stability of expression at different time points throughout the course of CLL represents an interesting area for future research. Clinical trials for assessment of therapeutic modalities targeting the *CRY-1* gene in larger cohorts of CLL patients are worthwhile.

## Figures and Tables

**Table 1:**
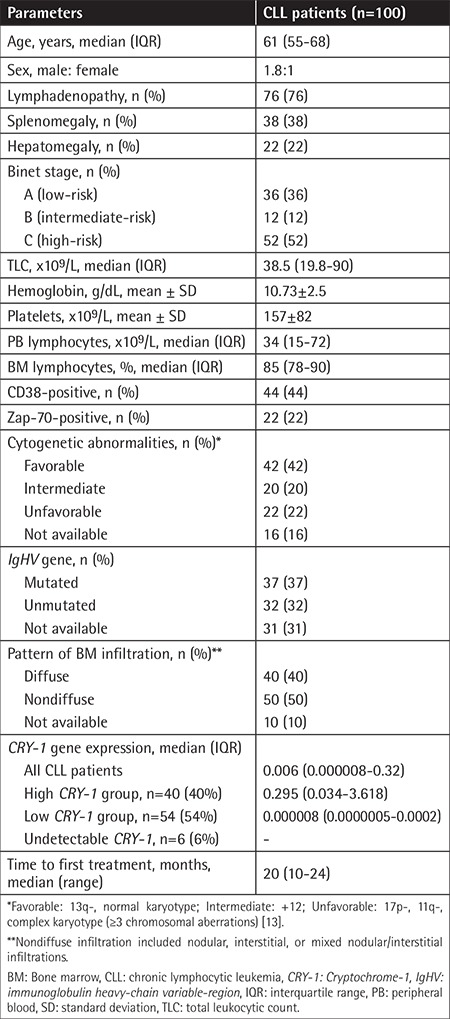
Clinicopathologic characteristics of chronic lymphocytic leukemia patients at diagnosis.

**Table 2:**
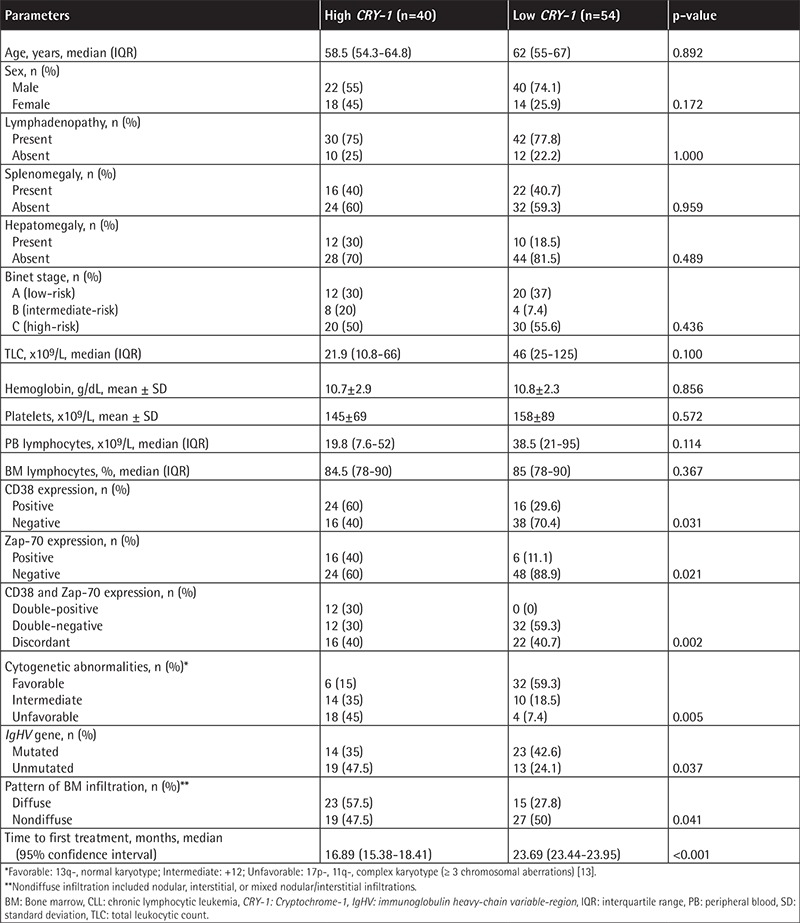
High and low *Cryptochrome-1* gene expression and clinicopathologic characteristics of chronic lymphocytic leukemia patients at diagnosis.

**Table 3:**
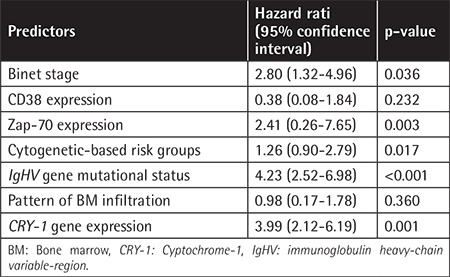
Predictors for earlier time to first treatment according to Cox proportional hazard regression model.
